# Comparison of soil microbial community between reseeding grassland and natural grassland in Songnen Meadow

**DOI:** 10.1038/s41598-020-74023-x

**Published:** 2020-10-09

**Authors:** Ruifen Zhu, Jielin Liu, Jianli Wang, Weibo Han, Zhongbao Shen, Taofeek O. Muraina, Jishan Chen, Dequan Sun

**Affiliations:** 1 Institute of Pratacultural Science, Heilongjiang Academy of Agricultural Sciences, 368 Xue Fu Road, Nangang District, Harbin, 150086 China; 2 National Hulunber Grassland Ecosystem Observation and Research Station, Institute of Agricultural Resources and Regional Planning, Chinese Academy of Agricultural Sciences, Beijing, 10008 China; 3 Department of Animal Health and Production, Oyo State College of Agriculture and Technology, P.M.B. 10, Igbo-Ora, Oyo State Nigeria

**Keywords:** Ecology, Plant sciences, Environmental sciences

## Abstract

Microorganisms have important ecological functions in ecosystems. Reseeding is considered as one of the main strategies for preventing grassland degradation in China. However, the response of soil microbial community and diversity to reseeding grassland (RG) and natural grassland (NG) remains unclear, especially in the Songnen Meadow. In this study, the soil microbial community compositions of two vegetation restoration types (RG vs NG) were analyzed using a high-throughput sequencing technique. A total of 23,142 microbial OTUs were detected, phylogenetically derived from 11 known bacterial phyla. Soil advantage categories included *Proteobacteria*, *Acidobacteria*, *Actinobacteria*, and *Bacteroidetes*, which together accounted for > 78% of the all phyla in vegetation restoration. The soil microbial diversity was higher in RG than in NG. Two types of vegetation restoration had significantly different characteristics of soil microbial community (*P* < 0.001). Based on a molecular ecological network analysis, we found that the network in RG had a longer average path distance and modularity than in NG network, making it more resilient to environment changes. Meanwhile, the results of the canonical correspondence analysis and molecular ecological network analysis showed that soil pH (6.34 ± 0.35 in RG and 7.26 ± 0.28 in NG) was the main factor affecting soil microbial community structure, followed by soil moisture (SM) in the Songnen meadow, China. Besides, soil microbial community characteristics can vary significantly in different vegetation restoration. Thus, we suggested that it was necessary and reasonable for this area to popularize reseeding grassland in the future.

## Introduction

Soil microorganisms, involved in various important ecological functions, are an important index for evaluating the response of various belowground biogeochemical processes of ecosystems to environmental changes^1–4^. However, ecosystem services and biodiversity have decreased by the human activities around the world^5,6^. Among the serious issues, the loss of soil microbial diversity is considered as the main threat to the balance of ecosystems. Moreover, the composition of soil microbial communities (SMC) varies widely across different biomes have been reported^2,7^. For instance, Zhang et al.^8^ found that the distribution of SMC is dependent on latitude gradient, while other studies revealed that it is also dependent on altitude gradient^9,10^, soil pH^7,11^, nutrients^12^, and temperature^13^. However, other factors like climate change, which currently disrupts the structures and functions of global ecosystems^14^, could also modulate SMC composition.

The Songnen Meadow of China is an ecologically fragile ecosystem vulnerable to various environmental disturbances due to its high altitude and extreme climatic conditions^9,15^. As climate becomes warmer and drier in Songnen Meadow area, together with increasing degeneration of permafrost, there is continuous decrease in soil surface water content, change in plant community, and/or significant succession, to the extent that the natural grassland has successively transformed into degraded grassland^16^. Consequently, Chinese Government launched the Grain-for-Grassland policy (or vegetation restoration project) largely contributed to maintaining soil and water conservation, which widely improved carbon sequestration and reduced floods^17^. For example, Yang et al.^18^ found soil carbon storage was closely related to soil bacterial diversity after vegetation restoration in Loess Plateau, and then Yang et al.^19^ confirmed that vegetation restoration greatly improved the physiological activity of soil microorganisms. Hence, according to the actual situation of the Songnen Plain, a lot of degraded grassland or abandoned farmland has been converted to reseeding grassland via natural succession. In fact, the vegetation restoration changes environment condition, by directly modifying root systems, and the exudates as well indirectly affecting soil quality and even soil microbes^20,21^. Concurrently, some studies have suggested that plant succession could significantly alter the structure of SMC and have a large-scale effect on soil carbon and nitrogen content^22,23^. Reseeding grassland is also considered as an effective measure for ecological restoration in this region^24^, which could restore ecosystem functioning and soil quality, but also influence soil microbe dynamics^25,26^. Therefore, a better understanding of the unique microbial geographical flora in this region will contribute to predictions of the impacts of vegetation restoration on ecosystems.

Previous research on vegetation restoration has mainly focused on vegetation coverage^27^, plant community structure^28^, plant primary productivity^29,30^, and soil carbon cycle or nitrogen cycle^31,32^. As a consequence, our knowledge regarding the structure of SMC in the ecosystem, particularly in the Songnen Meadow, and how the linkage between environment and microbial ecology may respond to the changing vegetation, is limited. With a growing body of biological evaluations at molecular levels, high-throughput sequencing technology has been widely and effectively applied in the research field of microbiology^33^. It can also be rapidly and effectively used to study the composition of microbial community and identify soil microorganisms via comparative analysis of total DNA. However, due to the present limitations in research techniques and analytical methods, few microbial diversity studies have thus far investigated the interactions between microbial species and population in the Songnen Meadow. Molecular ecological networks can provide important information about the complex ecological interactions between different species and reveal changes in the topological structure of microbial networks^34–38^. Statistical analyses showed that the approach was reliable, sensitive and robust in withstanding noise inherent in high-throughput data sets^39,40^. Hence, the molecular ecological network analysis method can be instrumental in predicting the microbial community composition^41^ and unraveling the microbial diversity response to different vegetation restoration.

Here, we investigated soil microbial community and diversity about two types of vegetation restoration (reseeding grassland and natural grassland) on the Songnen Plain. We hypothesized that soil microbial community characteristics have significant differences in different types of vegetation restoration and vegetation restoration strongly affects soil microbial community and diversity in this unique region.

## Materials and methods

### Sampling areas

**Figure 1:**
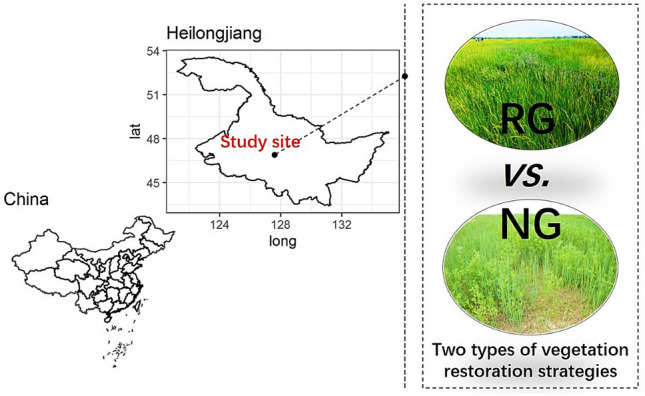
The study site is located in the Heilongjiang of northeast China. The paired meadows are selected after a long‐term vegetation restoration, including a reseeding grassland (RG) and a natural grassland (NG).

The experiment was carried out at the Frigid Forage Research Station located at Heilongjiang, China (Fig. 1), runned by Heilongjiang Academy of Agricultural Sciences (HASS). The station has an altitude of 160 m, longitude of 125°28′24″ E, latitude of 46°32′17″ N in Northeast China. The study site occupies a sub-humid climate. The mean annual temperature and precipitation are 5.3 °C and 425.5 mm, respectively. More than 75% of the annual precipitation occurred from June to August between 2008 and 2018. The topography of the area is typical for Songnen Meadow with elevations ranging from 160 to 165 m. The soil is a dark loam (mostly Chernozem, FAO Taxonomy) with high melanic humus^24^.

Songnen Meadow is facing an irreversible trend of degradation and there is now a total of ~ 200 hm^−2^ including both types of degraded and undegraded meadow. For this reason, both types of degraded and undegraded meadow has been used to evaluate different methods for vegetation restoration (Fig. 1). Degraded meadow characterized by hybrid grass needed reseeding methods for vegetation restoration in this region^42^, which are were selected to compare with natural grassland (undegraded meadow) in the effects of vegetation restoration on soil microbial community and diversity. The paired meadows, namely Dongsanbaishang (DSBS) and Xisanbaishang (XSBS), are now covered by different vegetation, respectively, but have similar soil, topography, and climate conditions (Table 1) and were cultivated and planted with forage crops before 2008.

**Table 1:** Site location, soil type and dominant species.

	RG	NG
Latitude	46°33.18′	46°26.87′
Longitude	125°56.95′	125°41.85′
Altitude (m)	160	165
Soil type	Meadow soil	saline-alkali soil
Dominant species	Medicago sativa, Leymus chinensis Tzvel, Lathyrus quinquenervius, and Stachys japonica	Leymus chinensis Tzvel, Carex duriuscula, Radix sanguisorbae, and Potentilla anserina L

*RG* reseeding grassland; *NG* natural grassland.

In DSBS meadows (0.636 km^2^, 1.2 × 0.53 km), reseeding grassland (RG) was adopted by reseeding forage crops (the primary reseeding species was *Medicago sativa*, and now the principal plant species belong to *Medicago sativa*, *Leymus chinensis* Tzvel, *Lathyrus quinquenervius*, and *Stachys japonica*) in 2008–2018, followed by prohibition of any cultivation and fencing until the present. There was no significant logging, grazing, or human activities. In XSBS meadows (0.33 km^2^, 0.6 × 0.55 km), natural grassland (NG) was adopted by natural grass restoration without human disturbance since 2008. Now the dominant plant species belong to *Leymus chinensis* Tzvel, *Carex duriuscula*, *Radix sanguisorbae*, and *Potentilla anserina* L. After 10 years of vegetation restoration and construction, these paired meadows have formed completely different vegetation landscapes, providing an ideal platform to examine the effects of different types of vegetation restoration on soil microbial community and diversity on Songnen Meadow. More information on soil type and doAppendix Aminant species can be found in Table 1. More details on belowground biomass, soil mechanical composition, soil physical and chemical properties between RG and NG can be found in .

### Experimental design and treatment description

We selected RG (*Medicago sativa*, *Leymus chinensis* Tzvel, *Lathyrus quinquenervius*, and *Stachys japonica*) in DSBS (reseeding forage vegetation) and NG (*Leymus chinensis* Tzvel, *Carex duriuscula*, *Radix sanguisorbae*, and *Potentilla anserina* L.) in XSBS (natural grass vegetation), with nine sample sites of each vegetation type. In each sample sites, we established three subplots located at least 100 m apart from each other. These subplots were a permanent quadrat (1 × 1 m) by a modified Whittaker plot. We gathered the surface soil samples (0–30 cm) in each subplot. Four replicates were obtained using a soil corer (6 cm in diameter) along an S-shaped curve, and then mixed to one sample. Additionally, we divided soil samples into two parts: one part was to analyze soil properties; for the other part was stored at − 80 °C immediately using liquid nitrogen for later DNA analysis.

### Sampling and laboratory procedure

#### Above-ground biomass

All samples were collected at the peak of biomass growing season (mid-August) in 2017. We estimated species-level cover, community-level cover, community-level above (ANPP) and below-ground biomass (BNPP) in one 1 m^2^ permanently positioned in each sampling unit. ANPP was annually clipped in each permanent quadrat. So, ANPP represents the total dry-weight biomass clipped at ground-level in each quadrat, including the grass and forb functional groups, which were then sorted apart (for further chemical analyses) before oven-drying at 65 °C for 72 h. Species diversity of the above-ground communities were estimated via Shannon–Wiener index (H) as described by Wang et al.^43^:

$$ H = - \Sigma Pi\ln Pi $$

where *i* is species, and *Pi* is the relative coverage of species *i*.

#### Soil physico-chemical properties

##### Physical properties

Soil samples were collected at the same time as plant samples. Soils in each plot were sampled annually via collecting three cylindrical soil cores of 30-cm depth and 5-cm diameter. Soil samples from the three different soil cores were thoroughly mixed s into one composite sample, and later divided into two subsamples. One subsample was stored at low temperatures for physical properties tests. The physical properties of the soil (including soil moisture, SM) were examined following the procedures described in Bao et al.^44^ (Appendix A). The other subsample was sieved (2-mm mesh) to remove roots and stones, and thereafter air-dried for chemical analyses.

##### Chemical properties

First, we determined soil organic carbon (SOC) and total N in the soil in each plot with the aid of FOSS Kjeltec 2300 Analyzer Unit (FOSS, Hillerød, Sweden). For total P, we followed the sodium hydroxide melting molybdenum antimony colorimetric method^18^. As described by Yang et al.^18^, soil available P (Olsen P) was obtained by shaking 1.5 g of dried soil for 30 min at 20 °C in 100 mL of a 42% NaHCO_3_ solution (pH 8.5). Finally, we determined the available N concentrations (AN) and NO_3_-N (ANN) in each plot after initial extraction of 10 g soil subsample with 50 mL of 2 mol/L KCl using a Flow-Solution analyzer (Flowsys, Ecotech, Germany).

### High-throughput soil sequencing

High-throughput soil sequencing was performed using a method described in Zhou et al.^45^. According to the overlap relation between paired end (PE) reads, the number of sample sequences in each stage was processed by statistical data, and the data quality was evaluated. Table 2 mainly counts the number of sequences in each stage, average sequence length, guanine and cytosine (GC) content, quality values of Q20 and Q30, Effective and other parameters. GC (%) is the percentage of G and C bases in the total bases. Q20 (%) is the percentage of bases with mass value greater than or equal to 20 in the total number of bases; Q30 (%) is the percentage of bases with mass value greater than or equal to 30 in the total number of bases; Effective (%) is the percentage of Effective Tags in PE Reads.

**Table 2:** Results of sample sequencing data evaluation.

	PE reads	Raw tags	Clean Tags	Effective tags	AvgLen (bp)	GC (%)	Q20 (%)	Q30 (%)	Effective (%)
RG	46,913	44,541	39,355	38,643	423	58.21	97.11	94.32	82.45
NG	66,551	63,212	56,108	54,992	419	56.28	97.24	94.65	82.61

### Network construction and analysis

Based on the results of high-throughput sequencing of soil bacteria^41^, Illumina HiSeq was used. The website of Institute of Environmental Genomics, University of Oklahoma (https://ieg2.ou.edu/MENA) was used to construct the molecular ecological network of the soil microorganisms with and without nitrogen application and to calculate its characteristic parameters. The construction process of the molecular ecological network is as follows: the operational taxonomic unit (OTU) data of soil microbial species were first converted into lg matrix, and then Pearson correlation moment was constructed and further converted into similarity matrix. According to the random matrix theory^39,40^, the adjacency matrix was derived from the similarity matrix by applying the appropriate threshold value, and the adjacency matrix was used to encode the connection strength between each pair of nodes. The characteristic parameters used for the description of the network information include: (1) fluidity, which is one of the most commonly used network indicators and in our study was used to calculate the total number of connections between each gene (node) and other connected genes, and to express the connectivity strength between a gene and other genes; (2) path length, that is, the shortest distance between two genes; (3) the clustering coefficient, which represents the degree of connectivity between one node and other nodes; and (4) the modularity of each network and description of the modularity of molecular ecological network. The ecological network was divided into several modules, and each module was a functional unit of the biological system. The function of node was represented by the connectivity within the module (*Zi*) and the connectivity between the modules (*Pi*). *Zi* measures the role of a point in all modules, and the higher the value, the greater the role in the module. Pi measures the degree to which a node participates in other modules. A higher value indicates a closer relationship with other modules. Cytoscape software was used to draw the network diagram and compare the structure of soil microbial network in the two alpine meadow types.

Linkages between network topology and environmental variables were also examined. Gene significance (GS) was defined as the square of Pearson correlation coefficient between relative abundance of genes in networks and environmental variables. Then, Mantel tests were used to measure the correlation between GS and connectivity^41^.

### Statistical analysis

All preliminary statistics and the different test data analyses were done with the SPSS 19.0 software. Microbial diversity was characterized by Shannon-Wienner and Simpson diversity indices, and microbial species richness was calculated. The characteristics of soil microbial community were sequenced by the detrended correspondence analysis (DCA). The relationship between community distribution pattern and environmental factors was estimated by the canonical correspondence analysis (CCA). Further statistical analysis was carried out using Vegan packages in R software; non-similarity test was analyzed using online websites (https://ieg2.ou.edu/MENA); SigmaPlot 12.0 was used for mapping and the molecular ecological network was visualized using the software of Cytoscape 3.0.

## Results

### Characteristics of plant in two types of vegetation restoration

The results showed that the dominant species in the RG were *Medicago sativa*, *Leymus chinensis* Tzvel, *Lathyrus quinquenervius*, and *Stachys japonica*. Plant height, cover, aboveground and underground biomass were significantly different in the two types of vegetation restoration (*P* < 0.05) with these parameters being higher in RG. The species richness of plant in NG was significantly higher than in RG (*P* = 0.001), but their Shannon–Wiener of plant diversity were similar (*P* > 0.05) (Table 3).

**Table 3:** Summary of plant characteristics in two meadow types.

	RG	NG	P
Height (cm)	71.51 ± 1.33	20.10 ± 0.73	0.000
Total coverage (%)	95.30 ± 9.63	32.70 ± 8.52	0.025
Aboveground biomass (g m^−2^)	677.80 ± 20.11	165.40 ± 13.60	0.000
Underground biomass (kg m^−2^)	85.10 ± 1.24	15.60 ± 1.05	0.000
Species richness of plant	9.30 ± 0.37	12.50 ± 1.38	0.001
Shannon–wiener index of plant	7.26 ± 0.25	8.13 ± 0.32	0.063

Significant test at *P* < 0.05.

*RG* reseeding grassland; *NG* natural grassland.

### Characteristics of soil in two types of vegetation restoration

The proportions of soil clay and silt were significantly different in the two types of vegetation restoration (*P* < 0.05), while the proportion of soil sand was similar (*P* > 0.05). Soil pH was significantly higher in NG than in RG (*P* < 0.05), and unlike soil moisture (SM). Soil total and available N concentrations, ammonium nitrogen, and soil total P concentrations were similar (*P* > 0.05) in the two types of vegetation restoration. Soil organic carbon and nitrate nitrogen were significantly higher in RG, while soil available P concentrations was higher in NG (*P* < 0.05) (Table 4).

**Table 4:** Summary of soil physicochemical characteristics in two meadow types.

	RG	NG	P
Clay (%)	2.19 ± 0.33	6.86 ± 1.08	0.000
Silt (%)	56.37 ± 1.55	50.58 ± 1.63	0.000
Sand (%)	41.42 ± 1.42	42.56 ± 1.37	0.053
pH	6.34 ± 0.35	7.26 ± 0.28	0.005
Soil moisture (SM)	37.62 ± 9.13	25.67 ± 8.37	0.000
SOC (g kg^−1^)	68.24 ± 11.38	59.86 ± 10.51	0.001
TN (g kg^−1^)	5.66 ± 1.31	5.38 ± 1.04	0.200
TP (g mg^−1^)	605.86 ± 38.46	664.75 ± 36.93	0.450
AN (g mg^−1^)	486.64 ± 44.37	407.35 ± 39.37	0.320
AP(g mg^−1^)	18.22 ± 2.18	43.67 ± 21.63	0.003
NH_4_^+^-N (g mg^−1^)	12.15 ± 3.33	6.88 ± 2.72	0.070
NO_3_^–^N (g mg^−1^)	43.56 ± 7.39	54.69 ± 8.42	0.010

Significant test at *P* < 0.05.

*RG* reseeding grassland; *NG* natural grassland; *SOC* Soil organic carbon; *TN* total nitrogen; *TP* total phosphorus; *AN* alkalize nitrogen; *AP* available phosphorus; *NH*_*4*_^*+*^*-N* ammonium nitrogen; *NO*_*3*_^*–*^*N* nitrate nitrogen.

### Differences in composition of soil microbial community

**Figure 2:**
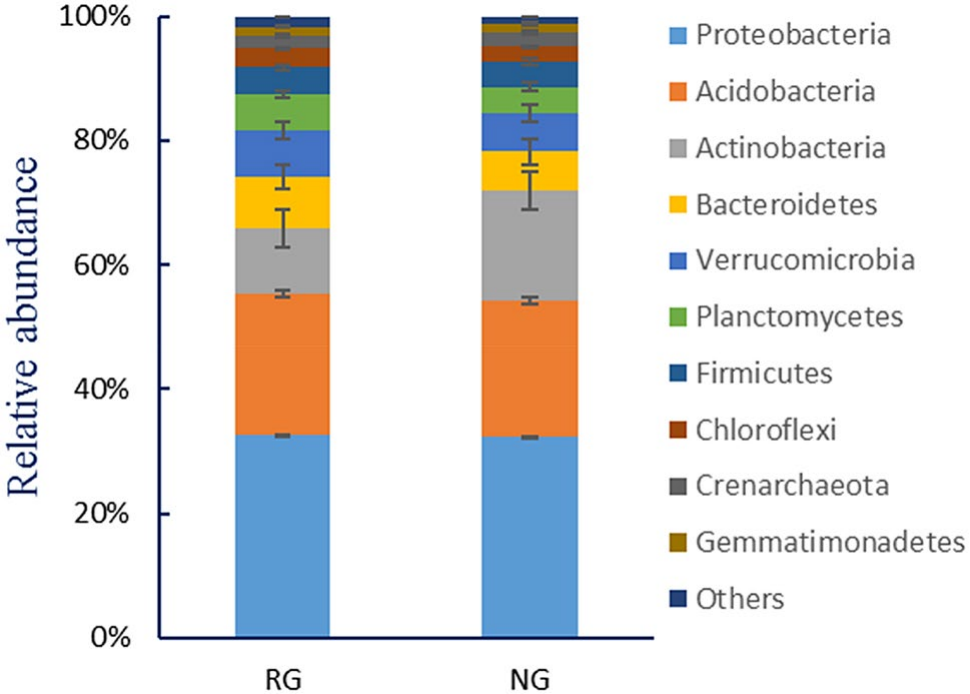
The relative abundance of soil microorganis in RG and NG at the level of bacterial phyla.

Detected OTUs can be divided into 32 kinds of bacteria and 2 categories of archaea, among which the *Proteobacteria*, *Acidobacteria*, *Actinobacteria* and *Bacteroidetes* were more abundant than others. The relative abundance of *Proteobacteria*, *Acidobacteria* and *Bacteroidetes* were significantly higher in RG than in NG, but *Actinobacteria* was lower in RG (*P* < 0.05). The relative abundance of soil microorgan in RG and NG at the level of bacterial phyla was shown in Fig. 2, respectively.

### Comparison of soil microbial diversity in two types of vegetation restoration

**Figure 3:**
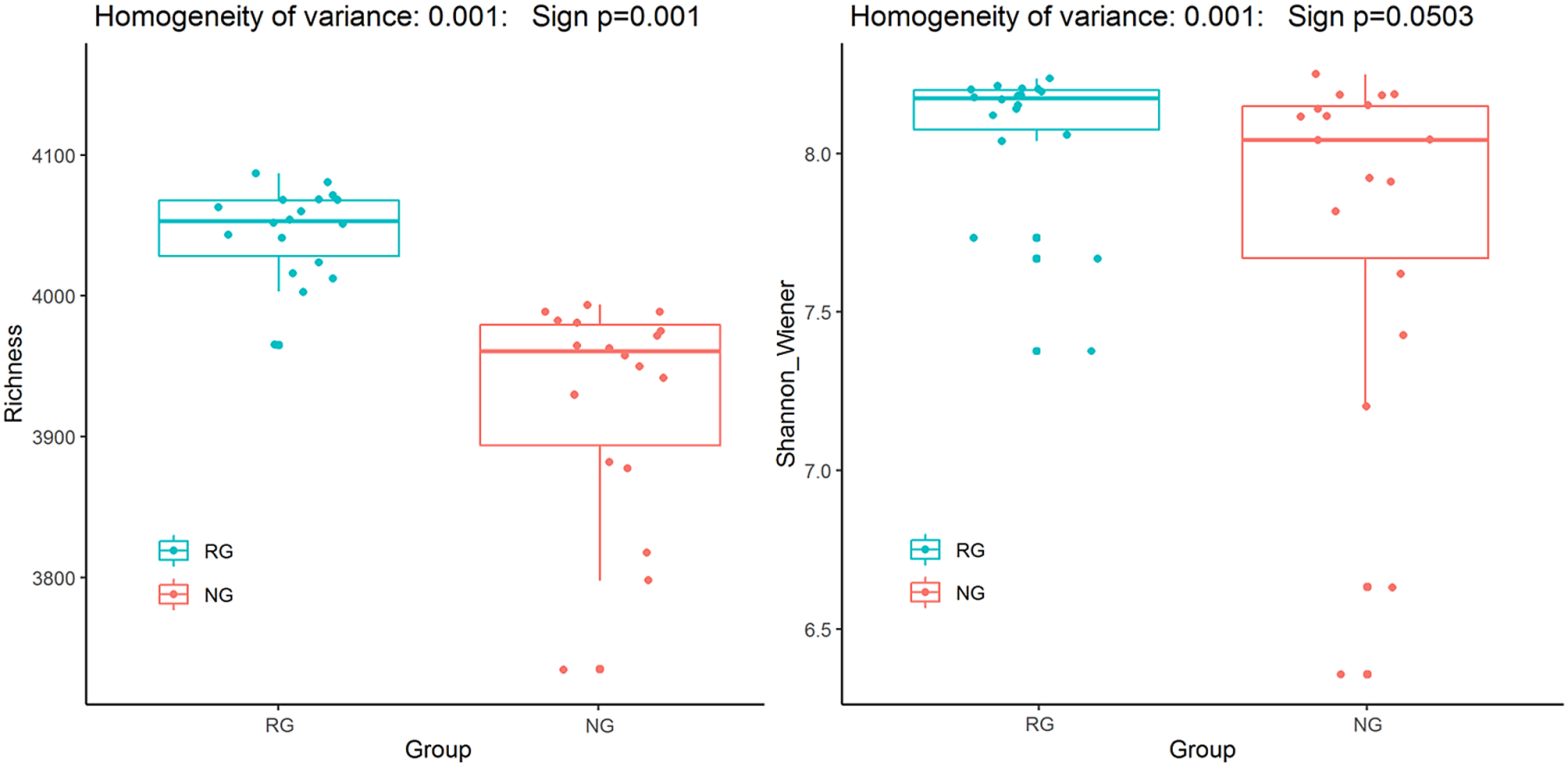
Diversity of soil microbial community in the reseeding grassland (RG) and the natural grassland (NG).

**Figure 4:**
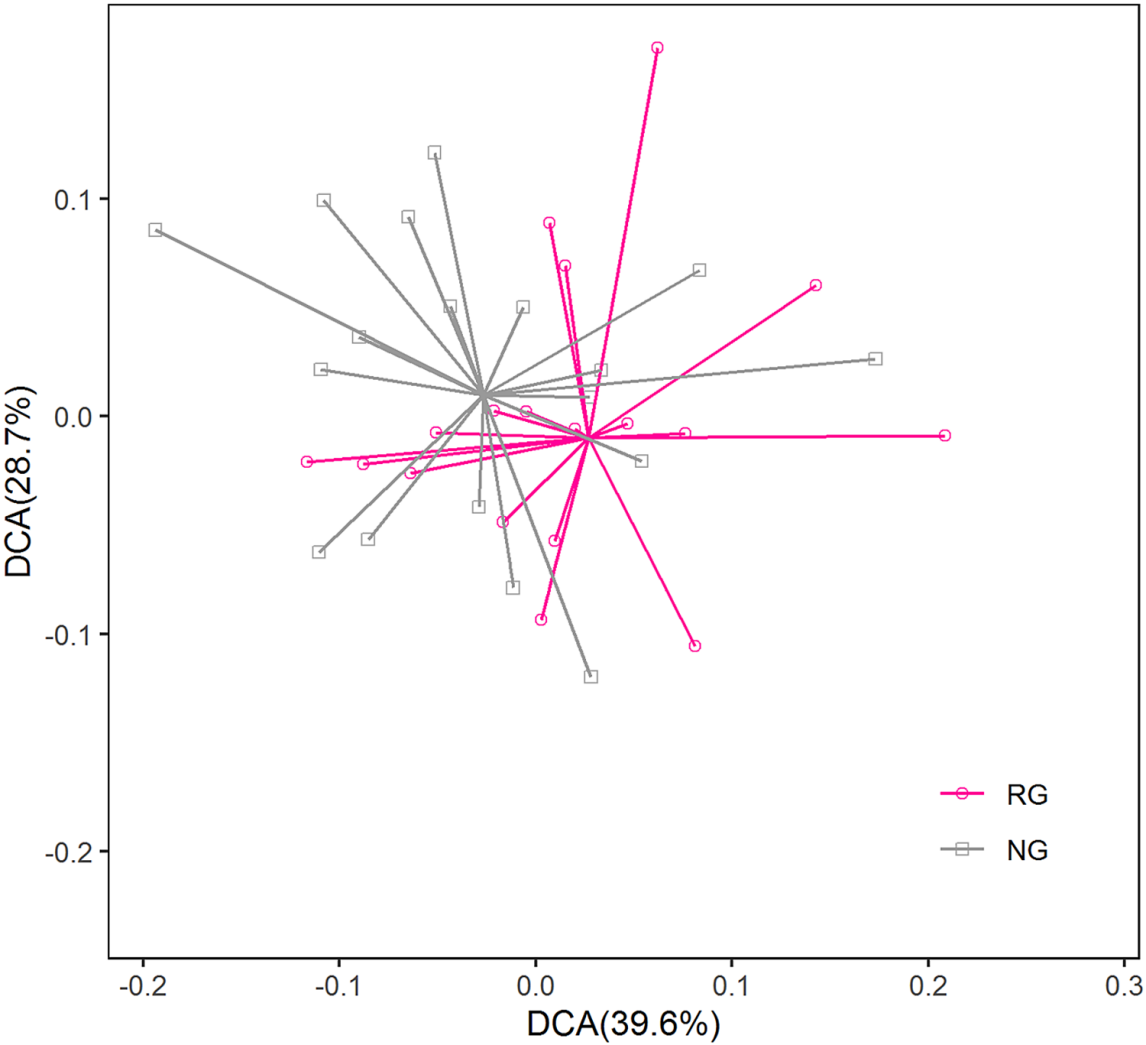
Detrended correspondence analysis of soil microbial community in the reseeding grassland (RG) and the natural grassland (NG).

The Illumina HiSeq was used for high-throughput sequencing and all soil samples were resampled according to 1500 sequences. Based on 97% similarity, a total of 23,146 OTUs were obtained, among which 15,547 OTUs were detected in RG and 16,236 OTUs were detected in NG. Although Simpson indices showed that the microbial diversity of the NG was significantly higher than that of the RG (*P* < 0.05), Shannon–Wiener showed that there was no significant difference between NG and RG (Fig. 3). The soil microbial community composition was sorted by the detrended correspondence analysis (DCA). As shown in Fig. 4, the soil microbial communities of the different vegetation restoration were basically separated. Further non-similarity tests (including MRPP, Adonis and Anosim) were conducted and the results also showed that the soil microbial community composition of the different vegetation restoration was significantly different (*P* < 0.05; see Appendix A).

### Molecular ecological network structure of soil microbial community

**Figure 5:**
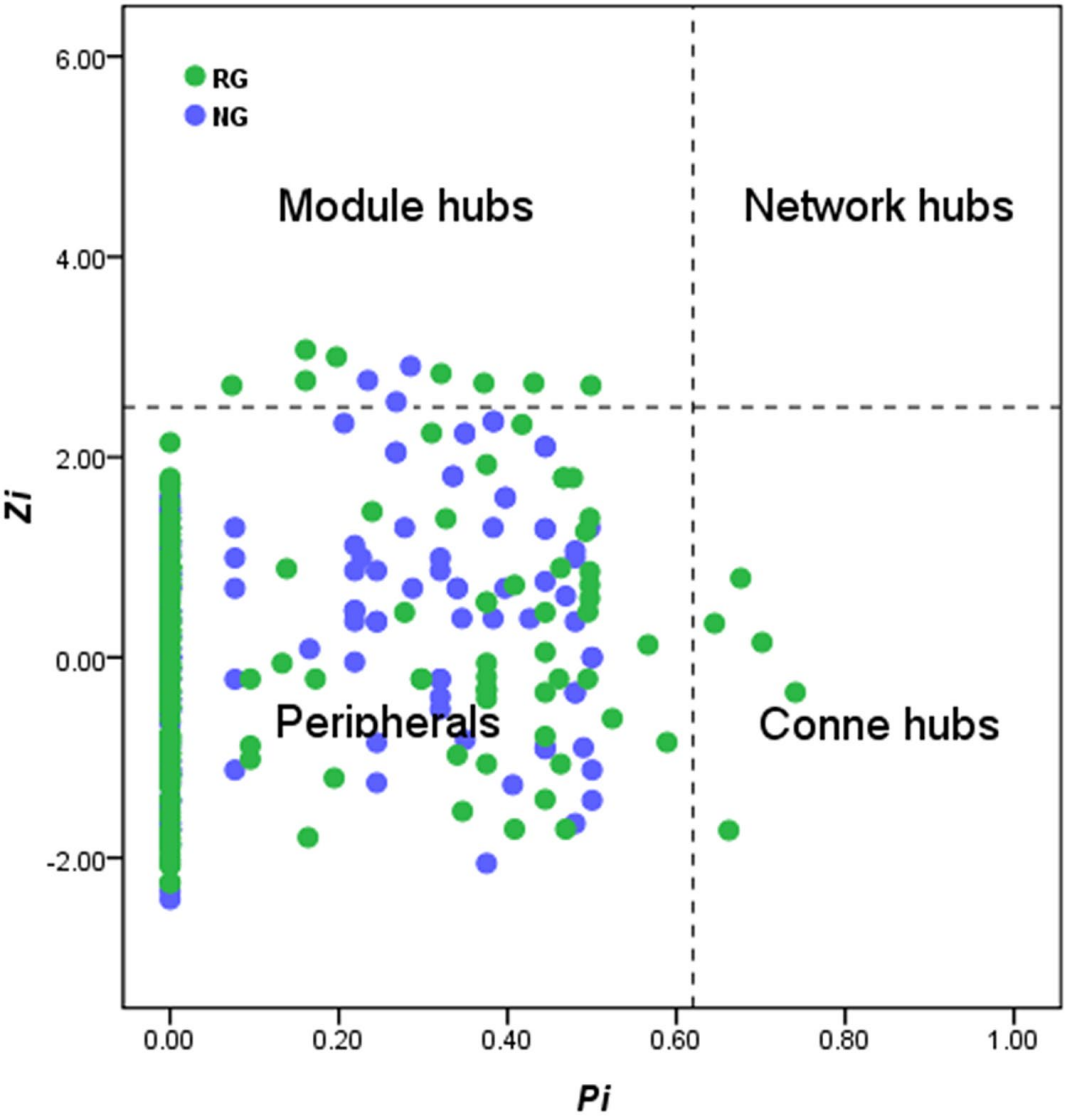
Topological properties of microbial networks in two types of vegetation restoration.

Based on the RMT algorithm, the network topological properties are shown in Table 5. The molecular ecological network of the two alpine meadow types have similar threshold, total nodes, total links, and modularity (*P* > 0.05). RG network has high connectivity, positive link percentage and average aggregation coefficient, while NG network has longer average distance (*P* < 0.05). As shown in Fig. 5, RG contains 8 module hubs, among which 5 and 3 module hubs belong to *Proteobacteria* and *Chloroflexi*, respectively, while NG contains 3 module hubs of *Actinobacteria*. RG contains 5 connectors, but NG has none.

**Table 5:** Topological properties of the empirical molecular ecological networks (MENs) of microbial communities in the RG andNG.

Types	Threshold	Total nodes	Total links	Positive link percentage (%)	Connectivity	Average path distance	Clustering coefficient	Modularity
RG	0.9	935	983	53.8*	0.43*	5.42*	0.137*	0.76
NG	0.9	963	895	49.6*	0.23*	6.99*	0.112*	0.81

*Representing a significant difference between RG and NG at *P* < 0.001.

### Environmental factors affecting the composition of soil microbial community

**Figure 6:**
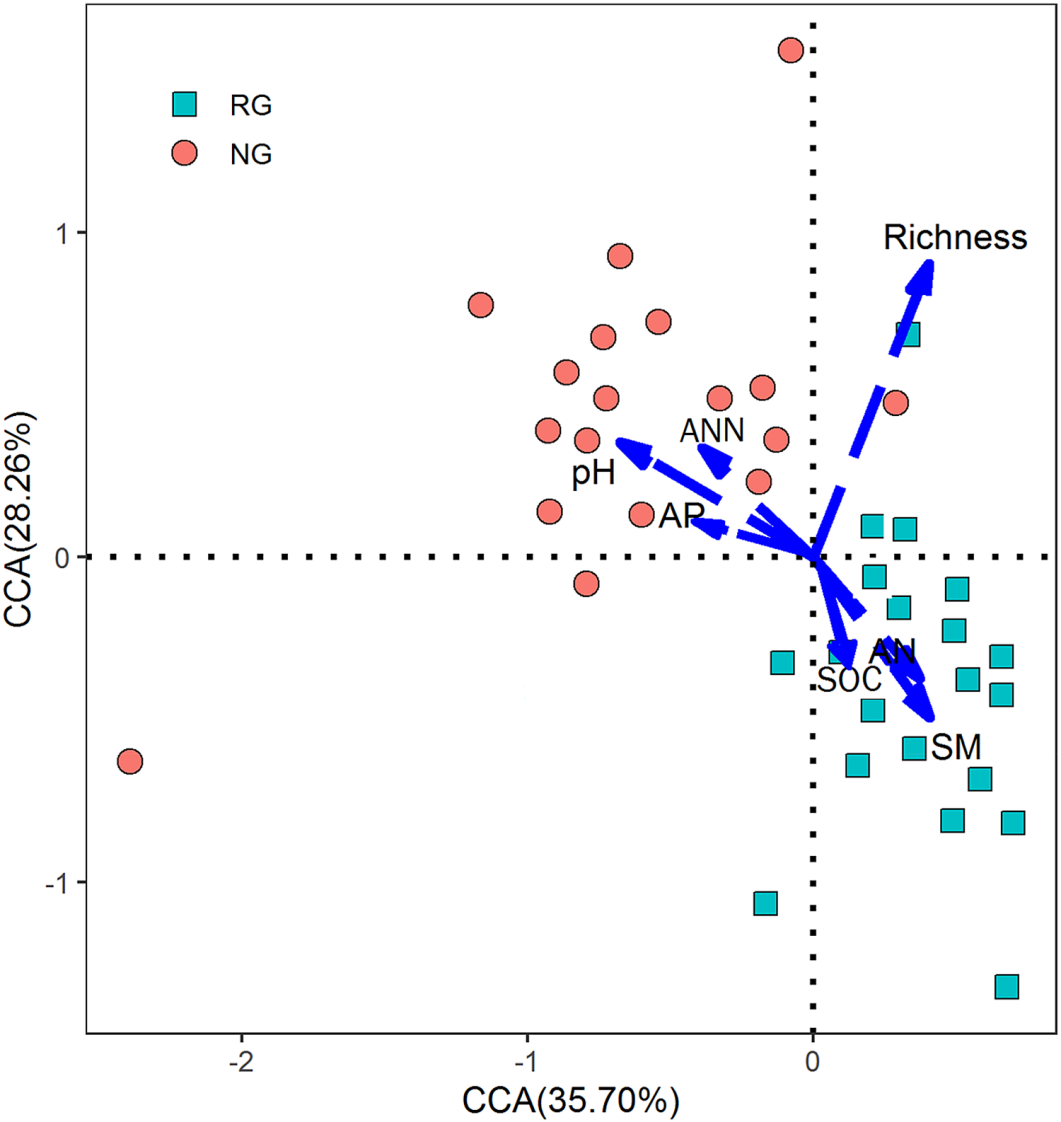
Canonical correspondence analysis of soil microbial community with environmental factors.

Canonical Correspondence Analysis (CCA) between environmental factors (SM, Richness, pH, soil available N, soil available P, soil organic carbon and NO_3_^–^N) and the composition of soil microbial communities was performed simultaneously as presented in Fig. 6. The arrows represent different environmental factors; the longer the Ray, the greater the influence of an environmental factor. An acute angle between the environmental factors means there is a positive correlation between the two environmental factors while an obtuse angle means a negative correlation. The cumulative contribution of all environmental factors combined is 63.96% and the first axis is 35.70% (Fig. 6).

The importance of environmental variables to network topology was examined as described previously^41,45^. First, gene significance (GS) was defined as the square of Pearson correlation coefficients between relative abundance of genes in a network and environmental variables. Then, Mantel tests were performed to examine the linkages between gene connectivity and GS, which showed that environmental variables were significantly correlated with network connectivity. In the network from RG presented in Table 6, node connectivity was significantly correlated with GS of pH (*P* = 0.005), soil moisture (SM, *P* = 0.015), soil available N (AN, *P* = 0.001), soil total organic carbon (SOC, P = 0.008), and aboveground plant richness (*P* = 0.050). In the network from NG, node connectivity was significantly correlated with GS of pH (*P* = 0.000), soil moisture (SM, *P* = 0.022), soil available N (AN, *P* = 0.000), soil available P (AP, *P* = 0.019), soil total organic carbon (SOC, P = 0.000), and aboveground plant richness (*P* = 0.020).

**Table 6:** Mantel tests on connectivity and GS of environmental variables.

Environmental variables	RG network		NG network	
	r	*P*	r	*P*
SM	0.035	0.015	0.043	0.022
Richness	0.018	0.050	0.019	0.020
pH	0.047	0.005	0.051	0.000
AN	0.033	0.001	0.029	0.000
AP	0.015	0.042	0.017	0.019
SOC	0.028	0.008	0.029	0.000
ANN (NO_3_^−^-N)	0.019	0.004	0.018	0.006

*RG* reseeding grassland; *NG* natural grassland.

## Discussion

Our findings clearly showed the response of soil microbial community and its diversity to two types of vegetation restoration (RG and NG) on the Songnen Meadow. We found these four groups of microbial as the dominant flora in the two experimental meadow types, accounting for > 78% of all the phyla. *Proteobacteria*, *Acidobacteria*, *Actinobacteria* and *Bacteroidetes* were more abundant than others (Fig. 2).These results are partly consistent with some studies. For instance, using high-throughput sequencing technology Shen et al.^46^ found that *Proteobacteria*, *Acidobacteria*, *Actinobacteria* and *Verrucomicrobia* are the main groups of microorganisms from Changbai mountain area, with relative abundance of 24.5%, 20.9% and 16.23%, respectively. A similar study by Zhang et al.^12^ on the changes of microbial community during the succession of alpine meadows also revealed *Proteobacteria*, *Acidobacteria*, *Actinobacteria* and *Bacteroidetes* as the dominant flora in the alpine meadow.

There was a higher Simpson indices and Shannon–wiener index in RG (Fig. 3), supporting our hypothesis that RG significantly affected soil microbial diversity, which confirmed by DCA ordinations and non-similarity tests (Fig. 3 and Appendix A). As far as we know, soil bacterial communities usually prefer different growth conditions and environmental conditions^47–49^. In our study, RG needs replanting plants and soil disturbance, which makes it easier to reduce soil bacteria and microbial diversity. On the contrary, there was no disturbance in NG, soil bacterial community rapidly increased in nondisturbed conditions, resulting in the higher soil bacterial diversity^8,50^.

Molecular ecological network analysis play an increasingly important role with the high-throughput sequencing technology providing a powerful information of microbial community composition^32^. In this case, RG network has high connectivity, positive link percentage and average aggregation coefficient, while NG network has longer average distance (p < 0.05) (Fig. 5). RG network contains 8 module hubs and 5 connectors, but NG network only has 3 module hubs without connector. These dominant microbial groups have the largest number of species, wide distribution, small individuals and strong diffusivity with which they form the characteristics of random and extensive distribution^51,52^. At the functional level in response to two types of vegetation restoration, unlike networks whose structures are already known, microbial networks are more complex and difficult to interpret^53,54^. To date, microbial interaction remains largely elusive^41^, while they can imply direct or indirect connections with ecological implication^55^. Based on the Random Matrix Theory Networks in our study, thresholds (all two thresholds were 0.900 in our study) were automatically chosen to avoid ambiguity in the network reconstruction^56^, so it renders networks more robust to noise^39,40^. From our network analyses, the shorter average path distance and the higher connectivity, which showed that the interaction between soil microbial is more complex in RG network than in NG network. Meanwhile, the shorter average path distance communicated to the entire network may be more sensitive to interference, so it means more stable in RG than in NG. In fact, NG network hub of module type is relatively singular, with most OTUs belonging to *Proteobacteria*, while the NG network is less resistant to the outside world because of the simplifying community composition^57^. Our results also were confirmed by this report that network modularity can be used as a resistance indicator of network system against external changes^58^.

Soil pH is usually one of the important factors that affect microbial diversity, and it has a close relationship with soil microbial community structure and composition^59^. Our results indicated that pH was the main factor determining soil microbial community features, followed by soil moisture (SM) in the Songnen meadow of Northeast, China. These results are partly consistent with some studies. For instance, Shen et al.^11^ found that soil pH controls soil microbial diversity and community composition and concluded that soil pH can affect the distribution of microbial widespread influence. Zhang et al.^1^ also found pH to be the main factor affecting SMC structure and the magnitude of microbial distribution patterns. Based on the method of constructing network analysis, soil pH value had the highest connection in the network interactions between environmental factors and microbial taxonomic community OTUs. Thus, it was not difficult to understand that Soil pH and soil moisture may have indirect influence on other soil physical and chemical properties^60^, understanding its potential relationships may be an important question for future research to improve our predictability of the vegetation restoration effects on terrestrial ecosystems.

## Conclusions

Overall, our finding underscores the importance of soil microbial community structure and diversity in different vegetation restoration (RG *vs*. NG) on the Songnen Meadow. There were different molecular ecological networks of vegetation restoration, which were characterized as scale-free, small-world, modular and hierarchical. The RG network has a longer average path distance and higher modularity, which make it more resistant to external environmental changes, including climate variability, than the NG network. Consistently, soil microbial community characteristics are significantly different across vegetation restoration. The composition of soil microbial community change is important for evaluating the responses of ecosystems to vegetation restoration. This will be special use for anywhere to improve vegetation restoration in further.

## Supplementary information

Supplementary Information.
