# Supplementary material for: Comparison of soil microbial community between reseeding grassland and natural grassland in Songnen Meadow
Source: Sci Rep. 2020 Oct 9;10:16884. doi: 10.1038/s41598-020-74023-x (PMC7547709; doi:10.1038/s41598-020-74023-x)
Supplement: Supplementary file 1 — Supplementary Information. [file 41598_2020_74023_MOESM1_ESM.docx]

**Appendix A :**

**Comparison of soil microbial community between** **reseeding grassland and**

**natural grassland in Songnen Meadow**

Ruifen Zhu^1^, Jielin Liu^1^, Jianli Wang^1^, Weibo Han^1^, Zhongbao Shen^1^, Taofeek O. Muraina^2,3^, Jishan Chen^1*^ and Dequan Sun^1*^

^1^Institute of Pratacultural Science, Heilongjiang Academy of Agricultural Sciences, Harbin, 150086, China;

^2^National Hulunber Grassland Ecosystem Observation and Research Station, Institute of Agricultural Resources and Regional Planning, Chinese Academy of Agricultural Sciences, Beijing 10008 China;

^3^Department of Animal Health and Production, Oyo State College of Agriculture and Technology, P.M.B. 10, Igbo-Ora, Oyo State, Nigeria.

**Further different information between RG and NG can been found in Appendix A.**

Table 1 Comparison of soil mechanical composition in different soil layers

|  | Soil layers(cm) | Soil mechanical composition**（%）** | | |
| --- | --- | --- | --- | --- |
|  |  | Clay  (<0.002mm) | Silt  (0.002-0.05mm) | Sand  (0.05-1mm) |
| RG | 0-10 | 4.09±0.68 | 67.47±1.10 | 28.44±0.83 |
|  | 10-20 | 1.73±0.27 | 62.08±2.08 | 36.19±1.94 |
|  | 20-30 | 0.77±0.04 | 39.57±1.47 | 59.63±1.51 |
| NG | 0-10 | 8.54±0.81 | 54.24±1.71 | 37.22±1.04 |
|  | 10-20 | 6.36±0.80 | 50.09±1.20 | 43.55±2.00 |
|  | 20-30 | 5.69±1.63 | 47.41±1.99 | 46.90±1.07 |

RG: reseeding grassland; NG: natural grassland

Table 2 Comparison of soil physical and chemical properties between RG and NG

|  | Soil bulk density  (g/cm3) | OM  (g/Kg) | TN  (g/kg) | TP  (g/kg) |
| --- | --- | --- | --- | --- |
| RG | 1.126±0.063 | 49.050±1.297 | 2.423±0.266 | 0.488±0.091 |
| NG | 1.452±0.023 | 21.242±2.364 | 1.075±0.214 | 0.260±0.077 |

RG: reseeding grassland; NG: natural grassland

Table 3 Dissimilarity test of microbial community composition between RG and NG

| Dissimilarity test | δ/R/R^2^ | P |
| --- | --- | --- |
| MRPP | δ= 0.634 | ＜0.001 |
| Anosim | R = 0.672 | ＜0.001 |
| Adonis | R^2^= 0.386 | ＜0.001 |

RG: reseeding grassland; NG: natural grassland
